# Integrating science and human values for cancer patient care

**DOI:** 10.3747/co.v15i0.268

**Published:** 2008-08

**Authors:** S.B. Sutcliffe

**Keywords:** Cancer, integrative care, science, evidence, values

## Abstract

The burden of cancer continues to increase globally, with substantial personal, societal, and economic consequences. Population growth and aging underlie this increase—a reflection of the effect of population health interventions in the last two centuries. Much of this gain has come through observation, derivation of evidence, and rigorous application of valid science to the public, both healthy and affected by diseases such as cancer. Increasingly, molecular medicine will affect the knowledge of cause and the personalization of therapy. However, science informs the decision-making process and places evidence within the beliefs of individuals and society as they relate to innovation, judgment, and values—the “logic” underlying alignment of conventional and complementary (holistic) care as a basis for compelling, consistent, and confident decisions.

## 1. INTRODUCTION

Integrative medicine brings together the care philosophies inherent in biomedicine—commonly conceived as Western, “evidence-based,” and rooted in science—and holistic medicine—often described in alternative or complementary medicine terms and based strongly in values or beliefs. Not uncommonly, this integration is obscured by debate as to which paradigm is “right” or “correct.” However, such a distinction is largely without value if the goal is to justify “care and decision-making” according to science (biomedicine) or to human values and beliefs (holistic medicine).

Science informs the process of decision-making and human values place scientific evidence within the context of the individual and society. In integrative care, not only are practices based in science and complementary medicine brought together, also brought together are the philosophies of analytic and deductive reasoning based in evidence (science) and the contextualization of science within the beliefs of individuals and society based on invention, judgment, and values (logic). A satisfactory outcome for the person needing care is confidence in the decision or decisions because information was made available, because the processes of assembling information were codified to allow for alignment with beliefs, and because the rationale for care formulated within the values of the individual was such that the eventual decision is compelling and consistent for that person.

## 2. DISCUSSION

### 2.1 Life, Death, and Human Values

**FIGURE 1:**
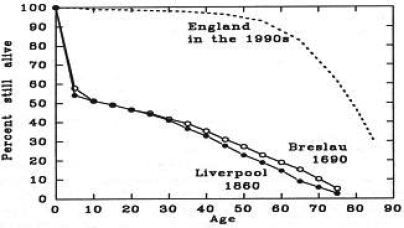
Patterns of survival in Breslau (17th century), Liverpool (19th century), and modern England (late 20th century). From Cairns 1997 1, p. 21, reproduced with permission.

In his treatise on the health of individuals and populations, Cairns notes that one cannot interpret health (life), without first being knowledgeable about illness and death 1. In Figure 1, attention is drawn to the unchanging pattern of human mortality from the earliest of available evidence to the mid-19th century, preceding the Industrial Revolution in the Westernized world. By contrast, the profound change in mortality following the Industrial Revolution (post-1850) is shown in the population survival plot for England in the 1990s (Figure 1). The illustration demonstrates certain points:

- The power of interventions to change survival—in this case, interventions largely related to the introduction of public health measures and, to a lesser, more recent degree, interventions based in medical treatment
- The value of observations validated through scientific methods as a basis for improvements in survival
- The factual basis for interventions designed to achieve longevity through informed, valid, applied health policy

In large part, interventions designed to control communicable diseases (pathogen A causes disease B, which can be controlled by intervention or interventions C) have resulted in avoidance of premature death, with resultant longevity. This benefit is based on a proposition that values longevity.

Through observation, deductive reasoning, intervention, validation through scientific method, analysis of outcome, and application to practice through policy, the population has, over one and one-half centuries, gained on average an extra 30 years of life expectancy from birth. However, in Westernized, high-income economies, cancer and most chronic diseases causing premature death are not communicable diseases for which straightforward interventions are available. Accordingly, the value set for communicable disease (longevity through avoidance of premature death) is not necessarily the same as that set for cancer or non-communicable diseases, in which functionality and quality determinants may take greater precedence than does longevity. Furthermore, the existing organization of health services based on an acute reversible illness model does not necessarily align well with the management of interventions to ameliorate the personal and societal impacts of chronic disease.

**FIGURE 2:**
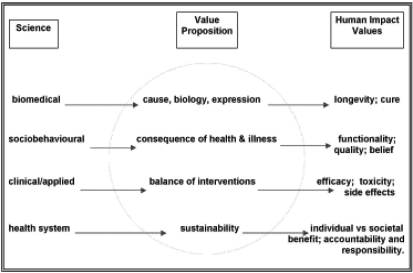
Domains of scientific enquiry.

In Figure 2, domains of scientific enquiry are related to effects on humanity according to the value proposition applied to the preferred outcome. The illustration draws recognition to the roles of individual values and societal values in determining the expected role of the intervention, thereby highlighting the dichotomy of the individual and society as consumer and beneficiary and funder of health care in a publicly funded health system.

### 2.2 The Cancer Control Problem

The population burden of cancer continues to increase, primarily as a function of the increase in population numbers and the structure of increasing age of our society. Cancer is principally a disease of aging, the rate increasing substantially after the age of 60 years. The rising burden of cancer draws attention to several issues:

- The effect of our interventions is insufficient to offset the increasing incidence consequent upon aging and population growth.
- The burden of cancer is not borne equally by individuals or populations: disparities exist within and across communities and populations.
- The interpretation and the effect of the burden has different meanings, all encompassed within the context of health need. That is,
  - “burden” as a statement of medically necessary care,
  - “burden” as a personal or societal consequence of illness,
  - “burden” as an expression of capacity to benefit from intervention,
  - “burden” as the capacity to afford necessary health care, and
  - “burden” as the expression of inequality to achieve the gains of intervention.

The interpretation of burden has both a personal and societal context and value proposition. Not-withstanding the definition or value placed on burden, the problem is not solely that the burden is rising—that is a fact. The problem is what can be done about the rising burden, individually and societally. A further point of importance is that the variation in health outcomes across communities and populations has as much, if not more, to do with variations in exposure to risk factors (environment, occupation, smoking, diet, obesity, nutrition, and exercise, among others) as with variations in access to interventional care.

### 2.3 Cancer As a Process, Not an Event

Molecular medicine has identified cancer as a genetic disease. Thus, the transformation of the healthy cell through a series of events that lead to a cancer cell with the properties of invasion and metastasis that may lead to death is underwritten in serial and cumulative changes within the genome of the cell. These are measurable biologic changes that correlate with predisposition, expression, sensitivity or resistance to therapies, and the prognosis of cancer—a concept underlying aspects of the uniqueness of cancer to the individual and to the personalization of care.

If we accept cancer as a process, arising in health and, if unperturbed, resulting in illness, disability, and death, then the strategy for cancer control must address the process, not just the disease. In the case of symptomatic cancer, the process is well advanced and the opportunities for intervention limited to treatment or palliation.

In the context of addressing the process of cancer, the definition and the nature of interventions related to cancer control assume importance 2. Cancer control encompasses

- cancer treatment: treatment interventions for individuals with an established diagnosis of cancer, commonly delivered through hospitals or cancer centres, focused on the treatment episode and the requirement for acute hospital-based services.
- cancer care: integrated programs across care settings (hospital, community, home) by networks of providers, with the goal of coordinated, continuous services for the individual or individuals experiencing cancer and needing care.
- cancer control: interventions directed to the healthy (at risk), those experiencing cancer, and those who are cured (survivors) or dying of their cancer. The interventions engage multiple sectors (health, education, and transport, among others) and are ultimately attempting to achieve a responsive, efficient, effective, and sustainable system to improve health and control cancer (as a process, not as an event).

Within each of these contexts of cancer intervention, values attached to the delivery of treatment, care, or control will be relevant both from an individual and a societal perspective. What ultimately drives the decisions regarding provision and access to interventions? How is this expressed from the perspective of the public, the patient, the care provider, and the funder?

Factors of relevance relate to the quality of the service (processes, outcomes), safety (incidents, errors, adverse events), accessibility (distance, costs), availability (timeliness), cost-efficiency and cost-effectiveness, satisfaction and sustainability (individually and societally), and attachment of individual and societal values and judgments to the foregoing parameters.

The premise underlying reductionism is that complex problems can be solved by dividing them into smaller—indeed individual—parts and by so doing, represent the whole by the sum of the individual parts. Scientific method would naturally lead us in the direction of the necessity to control all variation other than the characteristics and performance related to a single attribute or a small number of attributes. The current pursuit of targeted therapies is an illustration of that ideology, and it has been remarkably successful in a number of circumstances—for example, imatinib and tyrosine kinase inhibition in chronic myeloid leukemia and gastrointestinal stromal tumours; rituximab for CD20^+^ lymphoma; trastuzumab for breast cancer that is positive for human epidermal growth factor receptor. Yet notwithstanding these remarkable examples, reductionism has profound limitations as a strategy to explain and treat disease:

- It focuses on a single factor as cause.
- It emphasizes static control of function rather than dynamic management of a range of function.
- It implies a one-target, one-risk-factor approach to disease management.
- It takes a piecemeal approach to the multiple problems inherent in a chronic disease process.

Although inappropriately simplistic, reductionism aligns more to the management of communicable disease (pathogen A causes disease B, necessitating intervention C) than to the more complex gene–environment interactions characterizing non-communicable diseases including cancer.

Reductionism is a tool to investigate cause-and-effect through the scientific method, but other than in unique circumstances, it is incapable of addressing the whole simply as the sum of the parts within a system that is characterized by multiple biologic processes, multiple genes within multiple pathways, multiple targets, and multiple organ systems (tumour, tissue micro-environment, organ, host, and host environment). Even if a reductionist approach were to be employed to “dissect” the multiple, individual pathways underlying the cause and expression of cancer, would the establishment of therapeutic benefit through current clinical trial methods be feasible or practical? Current oncologic practice would suggest that only 5% of novel therapeutics actually enter clinical application, with an expected investment in excess of $800 million for each successful drug candidate achieving approval 3.

Challenges within the reductionist approach have stimulated the concept of a systems perspective to biology and health. In this construct, the holistic and composite characteristics of the problem are recognized, and the integration and interplay of relevant attributes are explored as an explanatory basis for observations. A relevant metaphor would be that “the forest cannot be explained by the study of individual trees.” The systems perspective would follow the principle that behaviour is explained by the system as a whole, not by the sum of the parts; that rarely is there a magic bullet for a unique, single target that will address health and illness; that many targets and many functions will be relevant to the control of biologic networks that include both cancer cells and normal cells; that time, space, and context will be relevant to how networks behave and respond; and that health is better defined by robustness, adaptability, and homeodynamism than by normalcy, control, and homeostasis 4.

### 2.4 The Concept of Integrated Health

Accordingly, how might health be viewed from a systems perspective?

A definition that would align to this concept would be “Health is the extent to which an individual or group is able, on the one hand, to realize aspirations and satisfy needs, and on the other hand, to change and cope with the environment. Health is, therefore, seen as a resource for everyday life, not the objective of living: it is a positive concept emphasizing social and personal resources as well as physical capacities” (cited in Young 1998 5).

This concept describes health in the context of functional potential: the individual as an asset whose function can be maximized and dysfunction minimized through planned mitigating interventions, recognizing that health risks (risk factors) are largely created and maintained by social systems, that the magnitude of risk exposure is a function of socio-economic disparities and psychosocial gradients, and that the development of sustained health or illness plays out over extended periods of time, crossing many stages of human development (prenatal to late adult, environmental, and socio-economic political domains) 6.

In this framework, disparities in health outcomes and in psychological adjustment to varying health states, rather than being attributable to discrete causes and actions, are determined by cumulative, compounding early-life events reflective of genes, biology, and environment embedded within the individual’s make-up, sustained by social, cultural, and economic forums, and affected by biologic, psychosocial, socio-economic, cultural, and physical environments. In each of these contexts, evidence established through the scientific method contributes to the development of a conceptual model for health and illness and the resultant response of health policies, practices, and systems 6.

## 3. CONCLUSIONS

Integrative care may be most aptly understood from the perspective of the beneficiary of care, the patient, rather than from the perspective of the care provider (the biomedical, complementary, or alternative medicine practitioner). In whose hands is the decision-making process placed? Which tools and information sources are made available to assist with the decision? How are the processes of reasoning codified—be they scientific, evidence-based, or debated—to provide a basis for decision-making that is aligned to individual beliefs? And how are arguments for interventions placed within the values and the priorities of the individuals?

Good decisions are made with the full confidence of the patient. Confidence requires the information to be sound, the rationale compelling, and the judgment to be fully aligned to patient needs and values. The inputs to the decision, be they scientific, holistic, complementary, or belief-based are all relevant inasmuch as trust in the inputs will lead to trust in the decisions.
